# A Comparative Analysis of the Photo-Protective Effects of Soy Isoflavones in Their Aglycone and Glucoside Forms

**DOI:** 10.3390/ijms131216444

**Published:** 2012-12-04

**Authors:** Barbara Iovine, Maria Luigia Iannella, Franco Gasparri, Valentina Giannini, Giuseppe Monfrecola, Maria Assunta Bevilacqua

**Affiliations:** 1 Department of Biochemistry and Medical Biotechnology, University of Naples “Federico II”, via S. Pansini 5, 80131 Naples, Italy; E-Mails: iovine@dbbm.unina.it (B.I.); marialuigia83@libero.it (M.L.I.); 2 Rottapharm-Madaus Dermo-Cosmetic R & D Division, Via Valosa di Sopra 9, 20052 Monza, Italy; E-Mails: info@gasparrifranco.it (F.G.); valentina.giannini@rottapharm.com (V.G.); 3 Department of Systematic Pathology, Section of Dermatology, Faculty of Medicine, University of Naples “Federico II”, via S. Pansini 5, 80131 Naples, Italy; E-Mail: monfreco@unina.it; 4 Faculty of Biotechnology Sciences, University of Naples “Federico II”, Via S. Pansini 5, 80131 Napoli, Italy

**Keywords:** isoflavones, cyclooxygenase-2, DNA-damage inducible gene, UVB, DNA damage, anti-inflammatory, photo-protective

## Abstract

Isoflavones exist in nature predominantly as glucosides such as daidzin or genistin and are rarely found in their corresponding aglycone forms daidzein and genistein. The metabolism and absorption of isoflavones ingested with food is well documented, but little is known about their use as topical photo-protective agents. The aim of this study was to investigate in a comparative analysis the photo-protective effects of isoflavones in both their aglycone and glucoside forms. In human skin fibroblasts irradiated with 60 mJ/cm^2^ ultraviolet B (UVB), we measured the expression levels of COX-2 and Gadd45, which are involved in inflammation and DNA repair, respectively. We also determined the cellular response to UVB-induced DNA damage using the comet assay. Our findings suggest that both the isoflavone glucosides at a specific concentration and combination with an aglycone mixture exerted an anti-inflammatory and photo-protective effect that prevented 41% and 71% of UVB-induced DNA damage, respectively. The advantages of using either isoflavone glucosides or an aglycone mixture in applications in the field of dermatology will depend on their properties and their different potential uses.

## 1. Introduction

Isoflavones are major soybean flavonoids that have recently attracted considerable interest because of their beneficial properties for coronary heart disease, cancer prevention, and osteoporosis [1]. In nature, isoflavones exist predominantly as glucosides such as daidzin and genistin and are rarely found in their corresponding aglycone forms daidzein and genistein [2,3]. Although all isoflavones are efficiently absorbed from the intestinal tract, there are striking differences in the fate of aglycones and beta-glucosides. After ingestion, the glucoside forms are hydrolyzed mainly by β-glucosidase of the intestinal microflora into aglycone forms that will be absorbed readily through the intestinal enterocyte [4]. Several studies have been conducted in humans to determine the relationship between the intake of isoflavones and their biological activity, including their absorption, distribution, metabolism, and excretion [2]. The effects of isoflavones differ depending on their glycosylation status [5]. In fact, aglycones are superior to glucosides in various bioactivities and are absorbed faster and in greater amounts than the glucosides in the human body [2,6]. Most studies thus far have evaluated the effect of isoflavones introduced by ingestion with food. Although the isoflavones are also recognized for their antioxidant properties in biological systems, there is little indication for their use as topical photo-protective agents. For example, topical application of equol, a daidzein metabolite, effectively reduced the incidence of cancers induced by chronic solar simulated ultraviolet (UV) radiation and provided UV-protective antioxidant effects [7]. Because the skin does not have the bacteria and enzymes required to process glucosides, preparations of isoflavones active for skincare are always in the form of aglycones. Previously, we investigated the photo-protective effects of different concentrations of genistein and daidzein in human skin cells irradiated with 60 mJ/cm^2^ UVB, both individually and in combination. We demonstrated that when administered in combination and at a specific concentration and ratio, genistein and daidzein exerted a synergistic photo-protective effect that was greater than the effect obtained from each isoflavone individually [8]. It was recently demonstrated that in addition to the aglycones, the isoflavone glucosides are also of physiological relevance. It was proved that glucosides genistin and daidzin were partly absorbed from the small intestine without previous cleavage and does not require hydrolysis to be biologically active [9]. Genistin also arrests the cell growth of human melanoma cells *in vitro* and inhibits UV light-induced oxidative DNA damage [10,11]. It was found that both genistin and daidzin exhibited a protective effect on DNA damage and exhibited a superoxide dismutase-like effect, but only genistin was able to significantly reduce the vitality of human melanoma cell lines, confirming the importance of the 5,7-dihydroxy structure in the A ring [9]. In addition, a preparation of an herb rich in isoflavone glucosides, such as genistin and daidzin from soya, stimulated the production of hyaluronic acid in normal human epidermal keratinocytes and thus could be used as a new cosmetic ingredient in moisturizers and antiaging agents [12]. In the present study, we performed a comparison analysis of the photo-protective effects of soy isoflavones in both their glucoside and aglycone forms using RPH-aglycone, a standardized glycine soy extract titrated to 90% isoflavone aglycones, in which the genistein is present in a defined 1:4 ratio with respect to daidzein (provided by Rottapharm-Madaus), as a source of aglycone forms. For this purpose, BJ-5ta skin cell lines were irradiated with 60 mJ/cm^2^ UVB, and we investigated the photo-protective effect of the glucoside isoflavones genistin and daidzin, both individually and in combination, and the RPH-aglycone, by testing several cellular parameters, such as cell vitality, cytotoxicity, and DNA damage. The UVB-induced DNA damage in cells was evaluated by the single-cell gel electrophoresis assay (comet assay). Anti-inflammatory and DNA repair properties were assessed by measuring the expression levels of COX-2 and Gadd45, which are involved in inflammation and DNA repair, respectively.

## 2. Results

**Figure 1:**
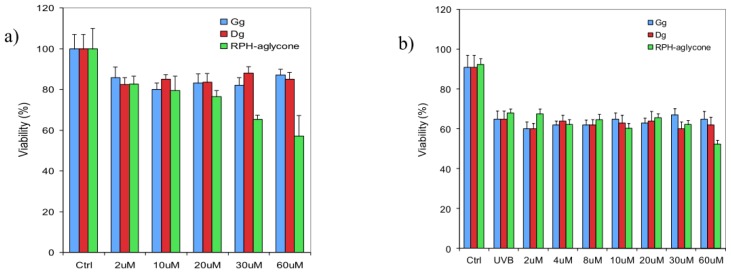
Effects of genistin, daidzin, and RPH-aglycone on the viability of unirradiated or UVB-irradiated skin cells. (**a**) The viability of unirradiated cells was measured with the MTT assay in BJ-5ta cells incubated either with genistin, daidzin or with RPH-aglycone (from 2 μM to 60 μM); (**b**) The viability of UVB-irradiated BJ-5ta cells was measured by Trypan blue staining 24 h after UVB irradiation in cells pretreated with genistin, daidzin, or RPH-aglycone. The results are reported as percentage cell viability, and the values represent the mean values ± SD of three independent experiments (Ctrl: untreated and unirradiated control cells; UVB: untreated and irradiated (60 mJ/cm^2^) cells).

In preliminarily experiments, we evaluated the effects of the soy isoflavones on cell proliferation and viability. For this purpose, we carried out MTT assays in proliferating BJ-5ta skin cells after treatment with increasing concentrations, from 2 μM to 60 μM, of isoflavones in the glucoside forms genistin and daidzin or in RPH-aglycone extract. BJ-5ta skin cells were collected for analysis after 24 h of treatment. As shown in Figure 1a, in unirradiated cells, neither the two isoflavones in their glucoside forms nor the RPH-aglycone extract affected cell viability at the concentrations tested. We then treated UVB-irradiated BJ-5ta skin cells either with genistin and daidzein or with RPH-aglycone using concentrations from 2 μM to 60 μM. At the concentrations tested, approximately 60% of the cells treated with genistin and daidzin, as well as those treated with RPH-aglycone, were still viable after irradiation with 60 mJ/cm^2^ (Figure 1b). In particular, 50% of BJ-5ta cells treated with 60 μM RPH-aglycone were still viable after irradiation. The samples were compared to untreated control cells, unirradiated control cells, and cells that were UVB-irradiated with a dose of 60 mJ/cm^2^.

**Figure 2:**
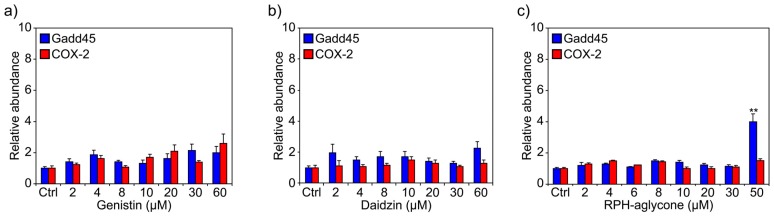
Effects of genistin, daidzin and RPH-aglycone on Gadd45 and COX-2 mRNA levels in unirradiated BJ-5ta cells. Levels of Gadd45 and COX-2 mRNA were determined by real-time PCR using a total RNA preparation from BJ-5ta cells treated with various concentrations of (**a**) genistin (from 2 μM to 60 μM); (**b**) daidzin (from 2 μM to 60 μM); or (**c**) RPH-aglycone (from 2 μM to 50 μM). The cells were harvested 24 h after treatment. The bars indicate the relative abundance of each mRNA; +1 is the abundance of Gadd45 and COX-2 mRNA in untreated cells. All values represent the mean ± SD of triplicate experiments. ^**^p < 0.001 (Ctrl: untreated control cells).

Subsequently, to determine whether the glucoside forms of isoflavones or the mixture of aglycone forms affected the mRNA levels of COX-2 and Gadd45, we examined gene expression by real-time PCR in non-UVB irradiated BJ-5ta skin cells. Figure 2 shows the results obtained after treatment with increasing concentrations of genistin or daidzin from 2 μM to 60 μM or with RPH-aglycone from 2 μM to 50 μM; cells were collected 24 h after treatment for analysis. In unirradiated cells, treatment with the glucoside forms did not affect the expression of COX-2 and Gadd45 (Figure 2a–c), but treatment with 50 μM of RPH-aglycone increased the mRNA level of Gadd45. These results were compared with a control sample of cells not treated with genistin, daidzin, or RPH-aglycone (Ctrl).

**Figure 3:**
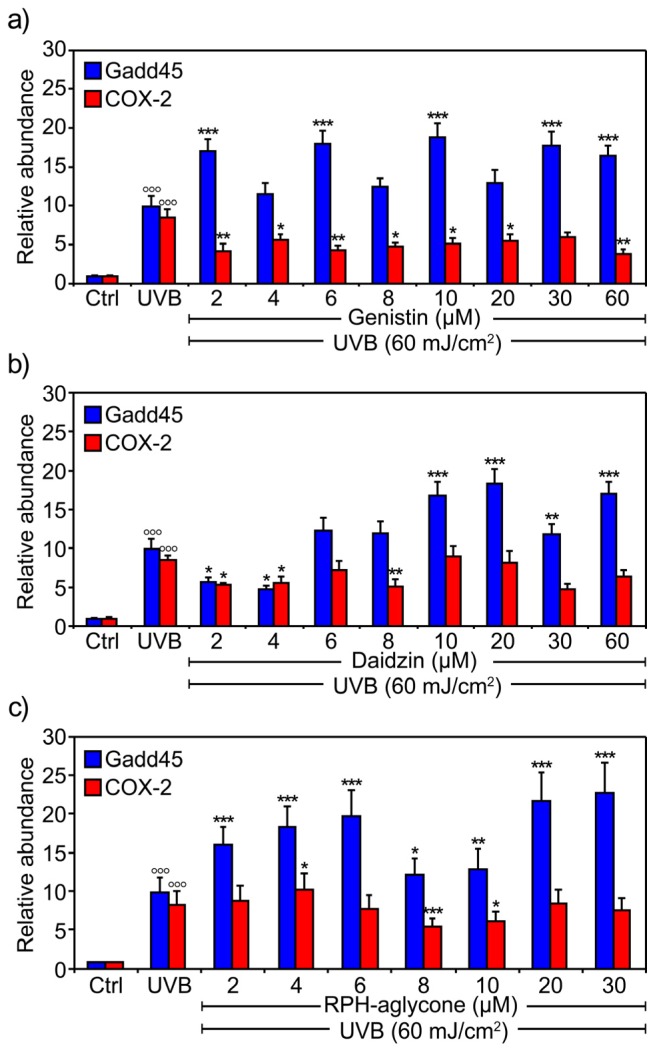
Effects of genistin, daidzin, and RPH-aglycone on the levels of Gadd45 and COX-2 mRNA in BJ-5ta cells irradiated with 60 mJ/cm^2^ UVB. Levels of Gadd45 and COX-2 mRNA were determined by real-time PCR in BJ-5ta cells treated for 2 h with various concentrations of (**a**) genistin (from 2 μM to 60 μM); (**b**) daidzin (from 2 μM to 60 μM); or (**c**) RPH-aglycone (from 2 μM to 30 μM). Cells were harvested 24 h after 60 mJ/cm^2^ of UVB irradiation. The bars indicate the relative abundance of each mRNA; +1 is the abundance of Gadd45 and COX-2 mRNA in unirradiated and untreated cells. All values represent the mean ± SD of triplicate experiments. ^*^p < 0.05, ^**^p < 0.001, ^***^p < 0.0001, ^○○○^*p* < 0.0001 (Ctrl: untreated and unirradiated control cells; UVB: untreated and irradiated (60 mJ/cm^2^) cells).

To test the effect of the isoflavone glucosides and aglycones on COX-2 and Gadd45 gene expression in BJ-5ta human skin cells irradiated with 60 mJ/cm^2^ UVB, we used a concentration of each isoflavone that did not induce gene expression in the unirradiated cells. Figure 3 shows the mRNA levels of COX-2 and Gadd45, as determined by real-time PCR, in BJ-5ta cells treated with concentrations from 2 μM to 60 μM of either genistin or daidzin or with RPH-aglycone concentrations from 2 μM to 30 μM 2 h before irradiation with 60 mJ/cm^2^ UVB. The isoflavone glucosides used singly at concentrations between 2 μM and 8 μM significantly reduced the mRNA levels of the COX-2 gene (Figure 3a,b). The effects of higher concentrations (from 10 μM to 60 μM) were not considered relevant for the combination treatment with both isoflavones. The Gadd45 mRNA expression level increased significantly after treatment with genistin (Figure 3a), and only treatment with 2–4 μM of daidzin reduced the Gadd45 expression level (Figure 3b).

Similarly, treatment with the RPH-aglycone mixture at 8 μM and 10 μM in irradiated cells reduced the level of COX-2 mRNA, but the expression of Gadd45 mRNA was not affected by treatment with these same concentrations (Figure 3c).

**Figure 4:**
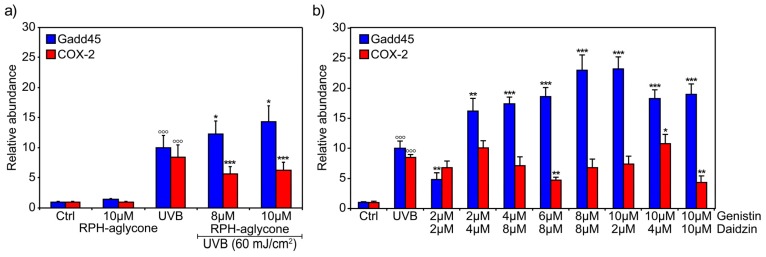
Effects of genistin and daidzin combinations and RPH-aglycone on the levels of Gadd45 and COX-2 mRNA in BJ-5ta cells irradiated with 60 mJ/cm^2^ UVB. Levels of Gadd45 and COX-2 mRNA were determined by real-time PCR using a total RNA preparation from BJ-5ta cells treated for 2 h with the most effective concentrations of (**a**) RPH-aglycone (8 μM to 10 μM) (**a**) or (**b**) with different combinations of genistin (from 2 μM to 10 μM) and daidzin (from 2 μM to 10 μM). The cells were harvested 24 h after 60 mJ/cm^2^ of UVB irradiation. The bars indicate the relative abundance of each mRNA; +1 is the abundance of Gadd45 and COX-2 mRNA in the unirradiated and untreated cells. All values represent the mean ± SD of triplicate experiments. ^*^p < 0.05, ^**^p < 0.001, ^***^p < 0.0001, ^○○○^*p* < 0.0001 (Ctrl: untreated and unirradiated control cells; UVB: untreated and irradiated (60 mJ/cm^2^) cells.

The results of the real-time PCR analysis at the most effective concentrations of the RPH-aglycone mixture are shown in Figure 4a. COX-2 expression levels were significantly reduced (*p <* 0.001) when the isoflavone glucosides were used in combination at lower concentrations (2 μM genistin and 2 μM daidzin) (Figure 4b). DMSO used as a vehicle control in non-UVB-irradiated BJ-5ta skin cells without isoflavones did not affect Gadd45 and COX-2 expression (Ctrl sample). The isoflavones at the concentrations tested, used either singly or in combination in unirradiated cells, did not affect cell viability (data not shown).

**Figure 5:**
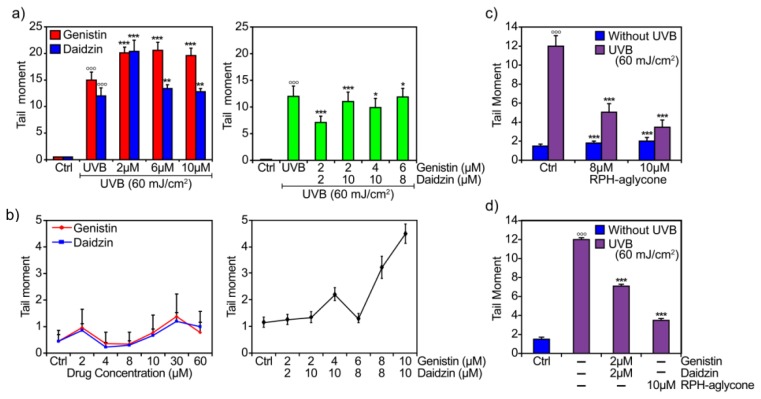
Analysis of UVB-induced DNA damage by the comet assay. (**a**) (left panel) BJ-5ta cells were treated for 2 h with different concentrations of genistin or daidzin (from 2 μM to 10 μM) before UVB irradiation and were harvested 24 h after UVB irradiation (60 mJ/cm^2^); (**a**) (right panel) BJ-5ta cells were treated for 2 h with different combinations of genistin and daidzin before UVB irradiation (60 mJ/cm^2^) and were harvested 24 h after UVB irradiation (60 mJ/cm^2^); (**b**) (left panel) BJ-5ta cells were treated for 2 h with various concentrations of genistin or daidzin (from 2 μM to 60 μM) and were harvested 24 h after treatment; (**b**) (right panel) BJ-5ta cells were treated for 2 h with different combinations of genistin and daidzin and were harvested 24 h after treatment; (**c**) BJ-5ta cells were treated with RPH-aglycone (8 μM and 10 μM) without UVB irradiation or before UVB irradiation (60 mJ/cm^2^) and were harvested 24 h after treatment; (**d**) A comparative analysis of the photo-protective effect by the comet assay of isoflavones in the aglycone (10 μM RPH-aglycone) or glucoside forms (2 μM genistin plus 2 μM daidzin) is shown. The comet assay procedure (see Experimental Section 3.7) was performed according to the manufacturer’s instructions. The results were quantified using NIH Image Software (version 1.62; NIH: Bethesda, MD, USA, 1997). The data are reported as TMs and represent the mean ± SD of three independent experiments. (Ctrl: untreated and unirradiated control cells; UVB: untreated and irradiated (60 mJ/cm^2^) cells).

Finally, we evaluated the cellular response to UVB-induced DNA damage using the comet assay. First, we assessed the effects of various concentrations of either genistin or daidzin glucosides (2 μM, 6 μM, and 10 μM) on UVB-induced DNA damage evaluated as Tail Moments (TMs). As shown in Figure 5a (left panel), treatment with genistin or daidzin 2 h before irradiation with 60 mJ/cm^2^ of UVB did not significantly prevent UVB-induced DNA damage. These concentrations did not affect DNA damage in unirradiated cells (Figure 5b, left panel). Subsequently, we examined the isoflavone combinations at concentrations of 2 μM, 4 μM, and 6 μM genistin in the presence of 2 μM, 8 μM, and 10 μM daidzin, respectively. The glucoside combination of 2 μM genistin in the presence of 2 μM daidzin most effectively protected against UVB-induced DNA damage, reducing DNA damage by approximately 41% (Figure 5a, right panel). Combinations that resulted in an increase in TM in unirradiated cells were excluded (Figure 5b, left panel).

Treatment with RPH-aglycone at concentrations of 8 μM and 10 μM 2 h before irradiation with 60 mJ/cm^2^ UVB reduced UVB-induced DNA damage by approximately 58% and 71%, respectively (Figure 5c). These concentrations did not induce DNA damage in unirradiated cells. The data reported were obtained with respect to untreated and unirradiated control cells. Figure 5d compares the results obtained with a combination of isoflavone glucosides (2 μM genistin plus 2 μM daidzin) to the best concentration of RPH-aglycone (10 μM).

## 3. Discussion

The major isoflavones in soybeans are the glucosides daidzin and genistin and their corresponding aglycone forms, daidzein and genistein. Their effects reflect their different states of glycosylation. The aim of this study was to investigate in a comparative analysis the photo-protective effects of isoflavones in their aglycone and glucoside forms. For this purpose, we investigated the efficacy of daidzin and genistin, either individually or combined, as well as an RPH-aglycone mixture (a standardized glycine soy extract titrated to 90% in isoflavone aglycones, with a 1:4 ratio of genistein to daidzein) in protecting BJ-5ta skin cells against the inflammation and DNA damage induced by UVB irradiation. First, we investigated the effect of the isoflavones in both their aglycone and glucoside forms on the cell proliferation and viability of BJ-5ta skin cells. We carried out trypan blue assays and MTT assays in non-UVB-irradiated and UVB-irradiated BJ-5ta skin cells after treatment with increasing concentrations of genistin, daidzin, or RPH-aglycone. We found that the addition of the glucoside forms did not significantly affect either cell viability or cell proliferation at any concentrations tested. Second, we investigated the anti-inflammatory and DNA repair properties of the isoflavones in their aglycone and glucoside forms. To this end, we evaluated the UVB-induced expression of COX-2 and Gadd45. COX exists in two isoforms, the constitutive COX-1 form and the inducible COX-2 form, both produced in abundance by activated macrophages and other cells at the site of inflammation [13]. The expression of COX-2 is generally undetectable under normal conditions but can be induced by various mitogenic and inflammatory stimuli including UV light [14]. In fact, UV radiation induces COX-2 expression to produce cellular responses, including aging and carcinogenesis in the skin [15]. Isoflavones showed strong inhibitory effects on the expression of COX-2 [16]. In particular, genistein has anti-inflammatory properties, inhibits UVB-stimulated prostaglandin E2 synthesis, and suppresses the UVB-induced expression of COX-2 in human epidermal cell cultures [17]. Gadd45 is a cell-cycle regulator and a DNA repair gene. The evidence accumulated so far suggests that the Gadd45 protein functions as a stress sensor, and Gadd45 has been implicated in stress-signaling responses to various physiological or environmental stressors resulting in cell cycle arrest, DNA repair, cell survival and senescence, or apoptosis [18]. In our study, the two isoflavone glucosides used singly at concentrations of 2–8 μM significantly reduced the expression level of the COX-2 gene (Figure 3a). On the other hand, the Gadd45 expression level increased significantly after treatment with genistin, whereas only low concentrations of daidzin (2–4 μM) reduced its expression level (Figure 3a,b). In addition, COX-2 and Gadd45 expression levels were both significantly reduced (*p* < 0.0001) when the isoflavone glucosides were used in combination at lower concentrations (2 μM genistin plus 2 μM daidzin) (Figure 4b). RPH-aglycone treatment in UVB-irradiated cells reduced the expression level of the COX-2 gene at concentrations of 8 μM and 10 μM but did not affect the expression level of Gadd45 at the same concentrations (Figures 3c and 4a). It is not surprising that the expression level of Gadd45 was not reduced, because its expression often indicates the presence of DNA damage as well as DNA repair [19]. Finally, DNA damage was analyzed using the comet assay, which is a sensitive method for detecting DNA strand breaks in a single cell and a versatile tool that is highly efficacious in human biomonitoring of natural compounds. In our study, the glucosides genistin and daidzin, administered singly 2 h before irradiation with 60 mJ/cm^2^ of UVB, did not significantly prevent UVB-induced DNA damage (Figure 5a). The glucosides used in combination with concentrations of 2 μM genistin and 2 μM daidzin, effectively reduced UVB-induced DNA damage by approximately 41% (Figure 5b). The RPH-aglycone mixture, administered at concentrations of 8 μM and 10 μM 2 h before irradiation with 60 mJ/cm^2^ UVB, reduced UVB-induced DNA damage by approximately 58% and 71%, respectively (Figure 5c). However, the interpretation of the data warrants several considerations. First, in nature, isoflavones exist predominantly as glycosides such as daidzin and genistin but only the aglycones have good transdermal absorption, although most recent studies report that also the glucosides can have strong transdermal activity [20,21]. Our findings show that, on UVB-irradiated skin cell lines, also genistin and daidzin, when administered combined and at a specific concentration and ratio, exert a synergistic anti-inflammatory and photo-protective effect. Second, in this study, we evaluated the effectiveness of a RPH-aglycone mixture, which is a standardized glycine soy extract titrated to 90% in isoflavones aglycones with a defined 1:4 ratio of genistein to daidzein. Therefore, we demonstrated that RPH-aglycone might also be considered as an active preparation of isoflavones that offers good protection against UVB-induced DNA damage in BJ-5ta skin cell lines. Our results are consistent with recent studies on the effects exerted by genistein and other isoflavones in combined formulations. Recently, it has been demonstrated that the topical application of solutions containing 0.5% of four individual isoflavones (genistein, daidzein, biochanin A and formononetin) photo-protects pig skin from either UV-induced sunburn cell formation and/or erythema The authors to investigate the mechanism of action, examine ethanolic solutions of isoflavones for their UV absorption and use the erythema response and sunburn cell numbers in pig skin to evaluate the photo-protective effect of isoflavone [22]. In addition, the isoflavone aglycone forms have poor solubility in water and oil; thus, a special galenic mixture is necessary to introduce these isoflavone preparations into cosmetic formulations. In a recent paper, the authors demonstrated that genistein generally exhibited greater skin absorption than daidzein [23]. However, daidzein permeation was enhanced when an aglycone mixture was used as an active ingredient [24]. In conclusion, this study reveals that isoflavones both in their aglycone and glucoside forms provide useful photo-protection, but probably by a different mechanism of action. The question is: what could be the potential benefit from their topical applications? Numerous experimental results indicate that the photo-protection can result from UV absorption of the topical solution or as a result of the antioxidant activity. Additional studies are needed to clarify their mechanism of action and to address whether the individual difference of percutaneous absorption among various isoflavones may account for the difference in protective efficacy.

## 4. Experimental Section

### 4.1. Cell Culture

BJ-5ta cells, which are human skin fibroblast cells immortalized with human telomerase reverse transcriptase, were cultured in a 4:1 mixture of Dulbecco’s medium (Gibco Laboratories, North Andover, MA, USA) and Medium 199 (Sigma-Aldrich, Oakville, ON, Canada) supplemented with four parts Dulbecco’s Modified Eagle’s Medium (Gibco Laboratories) containing 4 mM l-glutamine (Gibco Laboratories), 4.5 g/L glucose, and 1.5 g/L sodium bicarbonate and one part Medium 199 (Sigma) supplemented with 0.01 mg/mL hygromycin B (Sigma), 10% fetal bovine serum (Gibco Laboratories), and 1% penicillin/streptomycin (Gibco Laboratories). Cultures were maintained at 37 °C in a 5% CO_2_-humidified atmosphere.

### 4.2. Chemicals

Daidzin and genistin are glucosides that were supplied by Alfachem S.r.l. (Milan, Italy). RPH-aglycone was supplied by the Rottapharm-Madaus (Monza, Italy); this is a standardized glycine soy extract titrated to 90% in isoflavone aglycones, in which the genistein is present in a defined 1:4 ratio with respect to daidzein. The characterization of the aglycone solution in alcohol revealed that the product has a purity of >90%.

### 4.3. Treatment with Genistin and/or Daidzin or with RPH-aglycone and UVB Irradiation

BJ-5ta cells were plated onto 60-mm culture plates in 4 mL of fresh culture medium. After incubation for 1 day at 37 °C in 5% CO_2_, the samples were treated with genistin and/or daidzin and RPH-aglycone (dissolved in DMSO) 2 h prior to UVB irradiation at 60 mJ/cm^2^. Namely, the cells were treated with concentrations from 2 to 60 μM of genistin and daidzin, respectively, and from 2 to 50 μM of RPH-aglycone mixture. For different combinations, we used genistin in the presence of daidzin in the following formulations: 2 μM genistin plus 2 μM daidzin; 2 μM genistin plus 4 μM daidzin; 4 μM genistin plus 8 μM daidzin; 6 μM genistin plus 8 μM daidzin; 8 μM genistin plus 8 μM daidzin; 10 μM genistin plus 2 μM daidzin; 10 μM genistin plus 4 μM daidzin; 10 μM genistin plus 10 μM daidzin. After pre-treatment for 2 h with substances, the cells were washed and covered with 0.5 mL of phosphate-buffered saline (PBS), and the sub-confluent cells were irradiated with UVB (290–320 nm). The PBS was then replaced with 4 mL of culture medium containing again the isoflavones, and the cells were allowed to recover for 24 h. The control samples were treated with DMSO at the same concentration of different treatments and were processed as the other samples. Twenty-four hours after UVB irradiation, the cells were washed with PBS, and harvested to evaluate the DNA damage by Comet assay and to prepare total RNA for Real Time PCR analysis.

As a source of UVB, six Philips TL12/60W fluorescent lamps (Philips, Eindhoven, The Netherlands) emitting UVB light between 290 and 320 nm with a peak emission of 300 nm were used. The intensity of UVB irradiation, measured with a UV meter (Spectrolyne mod., Spectronics Corp., Westbury, NY, USA), was 0.8 mW/cm^2^.

### 4.4. Determination of Cell Viability

The MTT (3-[4,5-dimethyl-2-thiazolyl]-2,5-diphenyltetrazolium bromide) assay was used to measure cell viability after treatment with isoflavones. Briefly, 10 μL of MTT (0.05 mg/mL) was added into each well for additional 4 h incubation at 37 °C. After four hours, culture was removed and MTT formazan crystals were dissolved in acidified isopropanol (supplied by Sigma-Aldrich kit). Finally, the absorbance was measured spectrophotometrically with a microplate reader at a wavelength of 570 nm and background absorbance was subtracted measuring at 690 nm. The experiments were independently performed three times and each experiment contained triple replicates.

Cell vitality was also determined with the Trypan blue method [25]. Twenty-four hours after irradiation or after treatment with isoflavones, the medium was recovered, and the cells were washed twice with PBS and incubated with trypsin/EDTA. Cells were then harvested with the previously recovered medium and centrifuged at 1000× *g* for 10 min. The cell pellet was resuspended in an appropriate volume of PBS, and 0.5 mL of the cell suspension was combined with 0.5 mL of Trypan blue solution (supplied by Invitrogen, Life Technologies, Monza MB, Italy). The mix was incubated for 15 min at room temperature, and both the number of unstained cells (vital cells) and the total number of cells (vital and not) were determined on a hemacytometer under a microscope (dead cells take up the Trypan blue stain). The percentage of viable cells was determined by dividing the number of unstained cells by the total number of cells.

### 4.5. RNA Extraction and Reverse Transcription Polymerase Chain Reaction (RT-PCR)

BJ-5ta cells were exposed to UVB or treated with isoflavones for 2 h before UVB irradiation and harvested after 24 h for RNA extraction. RT-PCR was performed using total RNA. RNA was prepared with TRI Reagent, according to the manufacturer’s recommendations (Sigma Chemical Co., St. Louis, MO, USA). The purity of the RNA preparation was verified by measuring its absorbance ratio at 260/280 nm. Total RNA was subjected to cDNA synthesis with random hexanucleotide primers and MultiScribe reverse transcriptase (Invitrogen) at 48 °C for 1 h. The cDNA was then amplified in an iCycler iQ real-time PCR detection system (Bio-Rad Laboratories S.r.l., Segrate (MI), Italy) using iQTM SYBR Green Supermix (Bio-Rad Laboratories) in triplicate in 25 μL reaction volumes. Relative quantification of gene expression was performed using the 2^−ΔΔCt^ method. Actin served as the reference mRNA [26]. The ratios between 2^−ΔΔCt^ before UVB treatment and those calculated for the samples exposed to UVB light are expressed as fold changes. The primer sequences were as follows:

Gadd45 forward: 5′-AGACCCCGGACCTGCACT-3′
Gadd45 reverse: 5′-CCGGCAAAAACAAATAAGTTGACT-3′
COX-2 forward: 5′-CCTGGCGCTCAGCCATAC-3′
COX-2 reverse: 5′-GGTACAATCGCACTTATACTGGTCAA-3′
actin forward: 5′-CCTCACCCTGAAGTACCCCA-3′
actin reverse: 5′-TCGTCCCAGTTGGTGACGAT-3′

### 4.6. Single-Cell Gel Electrophoresis (Comet Assay)

DNA damage was evaluated with the comet assay as previously described [26]. Briefly, the cells were either exposed to UVB or treated with isoflavones 2 h before UVB irradiation, as described above, and 24 h later they were washed with PBS, trypsinized, resuspended in PBS, and combined with LM-agarose (supplied with the Trevigen kit; Trevigen Inc., Gaithersburg, MD, USA) at a ratio of 1:8 (cells:agarose). Electrophoretic and qualitative and/or quantitative analyses were carried out according to the Trevigen protocol [27]. The results were quantified using Image software, as suggested by the manufacturer. Data are reported as TMs, which is the ratio between the tail and nucleus areas.

### 4.7. Statistical Analysis

Results were expressed as the mean ± SE of 3 experiments. Statistical significance was calculated by one-way analysis of variance (ANOVA) and P value for a multiple comparison test (software GraphPad InStat 3.1; Dr. Harvey Motulsky: San Diego, CA, USA, 2012). The level of statistical significance was defined as ^*^p < 0.05, ^**^p < 0.001, ^***^p < 0.0001.

## 5. Conclusions

In summary, our study revealed that the soy isoflavones, in both their aglycone and glucoside forms, have an anti-inflammatory and a photo-protective effect on UVB-irradiated skin cell lines. Their potential benefit for topical application will depend on their use; however, the results of the present study may provide a basis for the use of the isoflavone glucosides and the RPH-aglycone mixture described herein as photo-preventive agents with promising applications in the field of dermatology.
